# Hormonal Regulation of Oligodendrogenesis I: Effects across the Lifespan

**DOI:** 10.3390/biom11020283

**Published:** 2021-02-14

**Authors:** Kimberly L. P. Long, Jocelyn M. Breton, Matthew K. Barraza, Olga S. Perloff, Daniela Kaufer

**Affiliations:** 1 Helen Wills Neuroscience Institute, University of California, Berkeley, CA 94720, USA; Jocelyn.Breton@nyspi.columbia.edu (J.M.B.); danielak@berkeley.edu (D.K.); 2 Department of Molecular and Cellular Biology, University of California, Berkeley, CA 94720, USA; mbarraza@berkeley.edu; 3 Memory and Aging Center, Department of Neurology, University of California, San Francisco, CA 94143, USA; olga.litvin@ucsf.edu; 4 Department of Integrative Biology, University of California, Berkeley, CA 94720, USA; 5 Canadian Institute for Advanced Research, Toronto, ON M5G 1M1, Canada

**Keywords:** oligodendrogenesis, hormones, mechanisms, steroids, peptides

## Abstract

The brain’s capacity to respond to changing environments via hormonal signaling is critical to fine-tuned function. An emerging body of literature highlights a role for myelin plasticity as a prominent type of experience-dependent plasticity in the adult brain. Myelin plasticity is driven by oligodendrocytes (OLs) and their precursor cells (OPCs). OPC differentiation regulates the trajectory of myelin production throughout development, and importantly, OPCs maintain the ability to proliferate and generate new OLs throughout adulthood. The process of oligodendrogenesis, the creation of new OLs, can be dramatically influenced during early development and in adulthood by internal and environmental conditions such as hormones. Here, we review the current literature describing hormonal regulation of oligodendrogenesis within physiological conditions, focusing on several classes of hormones: steroid, peptide, and thyroid hormones. We discuss hormonal regulation at each stage of oligodendrogenesis and describe mechanisms of action, where known. Overall, the majority of hormones enhance oligodendrogenesis, increasing OPC differentiation and inducing maturation and myelin production in OLs. The mechanisms underlying these processes vary for each hormone but may ultimately converge upon common signaling pathways, mediated by specific receptors expressed across the OL lineage. However, not all of the mechanisms have been fully elucidated, and here, we note the remaining gaps in the literature, including the complex interactions between hormonal systems and with the immune system. In the companion manuscript in this issue, we discuss the implications of hormonal regulation of oligodendrogenesis for neurological and psychiatric disorders characterized by white matter loss. Ultimately, a better understanding of the fundamental mechanisms of hormonal regulation of oligodendrogenesis across the entire lifespan, especially in vivo, will progress both basic and translational research.

## 1. Introduction

The human brain is able to undergo dramatic plasticity throughout life in response to both internal and external signals. Plasticity can include both structural changes, such as the formation of new synapses or changes to perineuronal nets, and functional changes, such as changes in network strength. Classically, neurogenesis, or the generation of new neurons, is regarded as a major form of plasticity [1]. However, the generation of new glia, or gliogenesis, is a lesser explored yet equally important avenue for investigation. Specifically, oligodendrogenesis, the creation of new oligodendrocytes (OLs), has emerged as a novel mechanism for experience-dependent plasticity in the developing and adult brain [2,3]. 

OLs are a class of glial cells in the central nervous system that produce myelin, a lipid-rich membrane that enwraps and insulates axons. Myelin is canonically known for its role in enhancing the speed of neuronal transmission [4]. However, OLs and their associated myelin have also been found to regulate plasticity. Specifically, myelin proteins inhibit axonal sprouting and are thought to close critical periods and crystallize circuits [5,6]. In the adult brain, myelin can undergo considerable reorganization in response to neural activity; this experience-dependent myelination ultimately contributes to motor function, spatial and motor learning, social behavior, and emotional affect [2,7,8,9,10,11]. 

**Figure 1:**
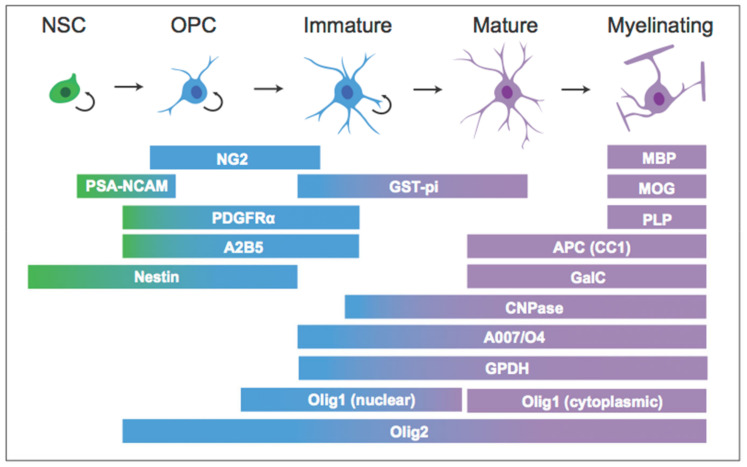
Cellular markers across the (oligodendrocyte) OL lineage.

Myelin plasticity in the central nervous system occurs via alterations to existing myelin, the addition of new myelin segments from existing OLs, and the incorporation of new OLs and myelin through oligodendrogenesis. Oligodendrogenesis occurs heavily during early development, yet also continues throughout adult life. As with neurons, OLs are originally derived from multipotent neural stem cells (NSCs) that maintain the capacity for self-renewal and can differentiate to adopt a neuronal or glial fate. These processes are tightly regulated by numerous factors, and the progression from NSC to mature OL can be tracked by the expression of characteristic cellular markers (Figure 1). For example, at the earliest stages, a subset of Nestin+ NSCs can develop into oligodendrocyte pre-progenitors (OPPs), proliferative cells that default towards a glial cell fate and express polysialylated-neural cell adhesion molecule (PSA-NCAM). A subset of OPPs become dedicated oligodendrocyte precursor cells (OPCs), precursors committed to the OL lineage; OPCs express a characteristic surface proteoglycan, neuron-glial antigen 2 (NG2), which allows them to be labeled and tracked in situ [12]. Other markers such as platelet derived growth factor receptor alpha (PDGFR-α) and the cell surface ganglioside A2B5 are also observed in OPCs and pre-oligodendrocytes [13]. The proliferation, survival, and differentiation of NSCs and OPCs can be quantified by tools that tag newborn cells, such as the synthetic thymidine analog bromodeoxyuridine (BrdU). Exogenously-administered BrdU is incorporated into DNA during the S phase, permanently labeling cells that were undergoing DNA replication at the time of BrdU administration. Hence, analysis of BrdU labeling soon after administration provides a means of quantifying proliferation. In addition, analysis of BrdU labeling at later time points allows for the quantification of newborn cell survival, as well as differentiation and fate trajectories through the use of double labeling with additional lineage markers. For example, a number of transcription factors, such as oligodendrocyte transcription factor 1 (Olig1), drive OPCs to differentiate into pre-OLs and immature OLs, and along with additional markers such as O4, Olig1 labels cells that have adopted an OL fate [13]. In contrast to Olig1, oligodendrocyte transcription factor 2 (Olig2) is expressed throughout the entire OL lineage [13]. At the final stages of maturation, OLs start to produce myelin and express markers unique to myelination, including myelin basic protein (MBP) and proteolipid protein (PLP) [13]. These markers are consistent across both developmental and adult oligodendrogenesis, and their detection is crucial for understanding the stages and regulation of oligodendrogenesis. It should be noted, however, that the exact delineations of the stages of oligodendrogenesis, e.g., when a cell is considered “immature” vs. “mature”, varies considerably across the literature, and the majority of studies only utilize one marker to define cell stage. A growing number of studies suggest that adult neural and glial precursors are heterogeneous populations, and many of the markers noted above can be expressed by cell types not of the OL lineage; for example, PSA-NCAM is also found in neurons and NG2 in pericytes [14,15]. Thus, future work should utilize multiple cellular markers or additional methods in order to be confident of cellular stage.

During mammalian development, OPCs originate from the ventricular germinal zones of the embryonic neural tube [16]. OPCs proliferate and migrate throughout the developing brain, and ultimately differentiate into mature, myelinating OLs. As with neurons, an overabundance of OLs is produced early in development. As these cells compete for growth factors, a portion ultimately undergoes apoptosis, resulting in a reduced population of myelinating OLs [17,18]. Although the production of myelinating OLs peaks in the first few weeks of life, there are regional differences in myelin maturation. For example, sensory areas myelinate earlier in life, while regions such as the prefrontal cortex extend myelination through adolescence and early adulthood [19]. Importantly, not all OPCs differentiate into OLs; a population of progenitors persist throughout the lifespan and retain the ability to proliferate and differentiate into OLs [20]. In addition, OPPs and OPCs can be produced from adult NSCs in the dentate gyrus and subventricular zone [21,22,23]. Importantly, OPCs are sensitive to neural signaling and respond to neural activity by proliferating and differentiating [24]. The survival and subsequent myelin production of these newborn OLs can ultimately contribute to the reorganization of the existing myelin landscape [2]. In addition, OPCs mobilize and differentiate into OLs in response to injury and demyelination, allowing for new myelination and repair [25,26,27]. Thus, oligodendrogenesis is a lifelong process in the central nervous system (CNS), with ultimate implications for development, circuit function, behavior, and various brain insults. Understanding how adult OPCs are regulated could lead to promising therapeutic avenues for demyelinating disorders such as multiple sclerosis. 

Oligodendrogenesis is a complex process, and the mechanisms that control oligodendrogenesis are under active investigation. OPCs and OLs are sensitive to numerous factors, including growth factors and a wide array of hormones [28,29,30,31]. Hormones, at their broadest definition, consist of a signaling molecule synthesized within an organism that acts upon an effector via a selective receptor. The distance a hormone travels and its method of transportation determine its signaling classification; for example, endocrine hormones are released into the bloodstream to act on distant tissues, whereas paracrine hormones are released locally to act on nearby cells [32]. Thus, endocrine hormones communicate to distant organs without the need for direct, neural innervation, and they have wide-reaching effects on an organism, from cognitive responses to stress to the homeostatic regulation of blood ion concentration [32]. The production of hormones also changes across development and at critical stages of life, including during puberty and whilst pregnant. Various hormones, including estrogens and stress hormones, alter neurogenesis in developing and adult mammals [33,34,35]. However, the role of hormones in gliogenesis, and in particular, oligodendrogenesis, is less well understood. 

In this review, we will explore how hormonal factors influence proliferation, differentiation, and survival across the OL lineage. We will restrict our discussion to the “classic” endocrine signaling molecules, which are typically released from a gland, travel through the bloodstream, and act upon distant tissues. However, several of the hormones discussed can also be produced by tissues in the CNS, thus acting in a paracrine fashion. This review will describe the effects of several major classes of hormones on oligodendrogenesis, including amino acid-based hormones (thyroid hormones, peptides, and amines) and steroid hormones (glucocorticoids and sex hormones). For each hormone, we will describe effects across the OL lineage in both development and adulthood, noting mechanisms where they are known. In a second, companion review [25], we discuss the implications of hormonal regulation of oligodendrogenesis for disorders characterized by alterations in oligodendrogenesis. We end with a discussion of future directions and additional considerations.

## 2. Amino Acid-Based Hormones (Thyroid Hormones, Peptides and Amines)

In this section, we discuss the role of amino acid-derived hormones and their receptors on oligodendrogenesis. These hormones can be genetically-encoded chains of two or more amino acids (peptides) or enzymatically altered compounds derived from single amino acids (amines and thyroid hormones). As a result, these hormones are typically stored in and secreted from vesicles and travel freely through the bloodstream, although some may be associated with binding proteins that aid in circulatory delivery and regulate bioavailability of the hormone. Because they are water-soluble, peptide and amine hormones typically act on cell surface receptors that utilize fast-acting second messenger systems [36]. In contrast, thyroid hormones are hydrophobic; thus, their mechanisms of action are more similar to steroid hormones than to peptides and amine hormones. For example, thyroid hormones primarily act through binding of nuclear receptors [37].

Below, we detail the roles of thyroid hormones, peptides (including insulin-like growth factor 1 (IGF-1), insulin, and prolactin), and the amine hormone melatonin in the regulation of the various stages of oligodendrogenesis. OLs have been shown through both transcriptional and histological studies to express the receptors for each of these hormones. Interestingly, while the downstream actions of these receptors are considerably heterogeneous, activation of many of these receptors, especially cell surface receptors, converge upon common signaling pathways, in particular the mitogen-activated protein kinase (MAPK)/extracellular signal-regulated kinase (ERK) and phosphatidylinositol 3-kinase (PI3K)/protein kinase B (AKT) signaling pathways. These pathways are broadly known to regulate cellular growth and survival [38,39], and indeed, although substantial gaps remain in our understanding of these hormones’ effects across the OL lineage, each of these hormones has been shown to enhance OPC proliferation and/or OL survival.

### 2.1. Thyroid Hormones

Thyroid hormones (THs) are tyrosine-based hormones that act on almost every cell type in the body to regulate CNS development and neuronal maturation, as well as overall organismal metabolism [40,41,42,43]. These two hormones, triiodothyronine (T3) and thyroxine (T4), are produced and released by the thyroid gland and are essential for the development and differentiation of cells, including OLs [44]. Their structures are based on the combination of two tyrosine amino acids that have been enzymatically modified to incorporate iodine molecules. T4 is the primary circulating TH, and enzymatic deiodination of T4 by type 2 deiodinase (Dio2) yields T3, the functionally active TH [40,41]. In the rat brain, Dio2 activity and corresponding levels of T3 increase after gestation and peak just prior to weaning [42,43,45]. THs cross the blood–brain barrier (BBB) into the CNS via various membrane transporter proteins [46]. 

Thyroid hormone receptors (TRs) are part of the nuclear receptor subfamily, and they bind either as homodimers or heterodimers to thyroid response elements (TREs) in DNA to alter gene expression [37]. In fact, TR binding to TREs occurs primarily due to heterodimerization with the retinoid X receptor (RXR) [47]. Interestingly, in the absence of TH or under conditions of low TH, unliganded TRs form a complex with co-repressor proteins, inhibiting gene transcription [37]. There are two primary classes of TR isoforms—TRα and TRβ—which are highly homologous but differ in their N-termini and their distribution across tissues in the body [48]. TRα and TRβ each produce several splice variants, three of which bind T3: TRα1, TRβ1, and TRβ2. Although nuclear receptors traditionally act at the level of transcription, TRs can also have non-genomic effects. For example, both TRα1 and TRβ2 can act via PI3K/AKT pathways to exert rapid effects [49,50]. Lastly, in addition to nuclear TRs, THs can bind to a transmembrane receptor, the integrin αvβ3 dimeric receptor, to exert non-genomic effects [51].

Nuclear TRs are expressed in OLs, both in vitro and in vivo [52,53,54,55]. Interestingly, expression differs across the OL lineage [56]. Specifically, OPCs express TRα, while differentiated OLs express both *TRα* and *TRβ* [52,53,57,58]. In addition, TRs dimerize with other nuclear receptors expressed in OLs [59], including vitamin D3 receptors, peroxisome proliferator-activated receptors, and the RXR [60,61,62]. Pre-myelinating, immature OLs from postnatal day (p) 0–2 rat pups also express the transmembrane receptor, αvβ3 [63], which regulates OL differentiation through a non-genomic pathway; in particular, binding αvβ3 activates PI3K/AKT and ERK pathways that help translocate TRs from the cytosol to the nucleus [64]. Together, expression of these receptors enables THs to affect oligodendrogenesis [65].

#### Effects on Oligodendrogenesis

At the earliest stages of the OL lineage, THs regulate proliferation, differentiation, and cell death of both developing and adult NSCs isolated from rodents. For example, TH is required for embryonic mouse NSC maintenance and proliferation; pharmacological depletion of TH inhibits proliferation, and TH binding to the αvβ3 receptor increases proliferation of cortical progenitors [66]. Excessive TH, however, can also have a negative effect on embryonic NSC proliferation [67], suggesting that there may be an optimal amount of TH for NSC proliferation. In addition to effects on proliferation, T3 also promotes embryonic NSC differentiation in vitro, promoting cells to differentiate toward a mixed glial fate [68]. Specifically, OPC quantities increase three-fold in the presence of T3 [69]. This effect requires the presence of the glycoprotein transferrin, which increases TRα1 expression [70]. Similar effects on oligodendrogenesis are observed in adult-derived NSCs. Consistent with the inverted U function of TH action, hyperthyroidism reduces NSC proliferation. Furthermore, treating adult-derived NSCs with T3 favors OPC differentiation [71,72]. Lastly, T3 acts not only on NSCs; treating OPPs (PSA-NCAM+) isolated from newborn rats with T3 enhances fibroblast growth factor 2 (FGF2)-mediated cell growth [68]. 

TH is also an essential hormone in modulating OPC proliferation and driving OPC differentiation. In particular, T3 prompts OPCs derived from developing rats to exit the cell cycle and differentiate into mature OLs [44,73,74]. The mechanisms of this switch are only partially elucidated and appear to depend in part on TRs [58,75,76,77]. T3 binding to TRα1 leads to complete arrest of OPC proliferation in vitro [78], and the absence of TRα1 results in continued proliferation of OPCs [76]. *TRα2* mRNA, which encodes a dominant-negative form of TRα, also decreases as OPCs proliferate, which may create a permissive state for TRα1 action and subsequent OPC differentiation [58]. Thus, several forms of TRα are involved in prompting OPCs to exit the cell cycle. TR-dependent effects on OPC differentiation are a bit more complex. Given that *TRβ* expression is confined to differentiated OLs, it is suggested that TRα receptors facilitate the effect of TH on OPC differentiation, while TRβ aids in terminal differentiation into mature OLs [58]. Indeed, overexpression of TRα accelerates rodent OPC differentiation in culture [77]. However, applications of TRβ agonists and TRβ overexpression also increase OPC differentiation; it is unclear if these effects are truly TRβ dependent or whether this exogenous upregulation of TRβ acts at the same TREs as TRα [58,75]. Importantly, these studies have all been conducted in vitro; in vivo studies will be necessary to confirm both the effects of TH on OPC development, and the role of specific TR variants. 

THs also induce OL maturation and alter OL morphology [71,79,80,81,82,83,84]. Specifically, THs promote both morphological and functional maturation of OLs through interactions with MBP promoter regions and transcriptional regulation of other genes such as myelin oligodendrocyte glycoprotein and glutamine-synthase [79,81,82,83,84]. Consistent with these findings, TH deficiency shortens the elongation process of mature OLs [80] and delays expression of MBP and 2’,3’-cyclic-nucleotide 3’-phosphodiesterase (CNPase), a myelin-associated enzyme [81]. T3 can also act in conjunction with 9-cis retinoic acid (which binds to RXR) to increase the number of pre-myelinating OLs, increase OL morphology complexity, and increase MBP expression [85,86]. Interestingly, in rodents, OL maturation is only influenced by T3 in the first three weeks of life after birth [87]. During that time, THs also enhance OL survival [88]. This effect may occur through TH’s ability to regulate survival-specific growth factors such as neurotrophin-3 and IGF-1 [89,90,91,92]. Outside of this time window, OLs no longer require TH to survive [88]. These findings suggest that TH may no longer have a role in OL maturation and survival in adulthood under physiological conditions. These differences in TH action across the lifespan are highly unexplored and are a fruitful area for future study.

Overall, THs potentiate oligodendrogenesis along the OL lineage, from specification of NSCs towards an OL fate, to cell cycle arrest and differentiation of OPCs, to maturation of immature OLs and increasing myelination in mature OLs. 

### 2.2. Insulin-like Growth Factor 1 (IGF-1)

IGF-1 is a 70 amino acid peptide that contributes to cell growth and proliferation, as well as cell survival [93,94,95]. This peptide is produced in the liver, and its secretion is stimulated by growth hormone (GH) and the GH receptors [96]. In the bloodstream, IGF-1 is largely bound to IGF-1 binding proteins [97]. In addition to hormonal delivery via the bloodstream, IGF-1 can be synthesized locally in the CNS by neurons and glia, including OLs [98,99].

While no studies to date demonstrate expression of GH receptors in OLs, all CNS cells, including OLs, express the IGF-1 receptor (IGF1R), a cell surface receptor with tyrosine kinase activity [94,100,101,102]. Ligand binding to IGF1R primarily induces the PI3K/AKT and the MAPK/ERK signaling cascades, which broadly inhibit apoptosis and promote mitogenesis, contributing to IGF-1’s function in cell survival and tissue maintenance [103]. IGF-1 can also bind with low affinity to the insulin receptor (IR) [104].

#### Effects on Oligodendrogenesis 

Overall, studies performed in vitro suggest that IGF-1 increases oligodendrogenesis by enhancing OPC survival, promoting OPC differentiation, and inhibiting apoptosis of developing OLs. At the earliest progenitor stages, IGF-1 promotes the survival of neonatal rat-derived PSA-NCAM+ progenitors in culture [105]. While IGF-1 alone does not promote proliferation of these cells [105], IGF-1 can act synergistically with growth factors such as epidermal growth factor and FGF2 to promote NSC proliferation [105,106]. In addition, IGF-1 biases NSCs towards an OL fate; specifically, IGF-1 greatly increases oligodendrogenesis from NSCs of the adult rat dentate gyrus, an effect driven by Noggin and SMAD family member 6 inhibition of bone morphogenic protein signaling [107]. 

Specifically in the OL lineage, IGF-1 dose-dependently promotes OPC survival in vitro through PI3K-dependent inhibition of caspase-3 (a crucial mediator of apoptosis) and subsequent cell death [17,108,109]. IGF-1 is therefore a potent survival factor for OPCs [108,110,111]. This effect persists past the proliferative phase of OL development, suggesting that IGF-1 promotes survival across the OL lineage [17,108].

While the anti-apoptotic effects of IGF-1 are well established, the mitogenic properties of IGF-1 on OPCs are less clear. Early in vitro experiments suggested that application of IGF-1 enhances proliferation of bipotential rat oligodendrocyte-type 2 astrocyte progenitor cells (O-2A, now commonly referred to as OPCs) [112]. Subsequent studies further demonstrated that IGF-1 induces [3H] thymidine and BrdU incorporation in neonatal rat cultured OPCs, indicating increased proliferation [113,114]. However, additional experiments have countered this, arguing that application of IGF-1 does not induce proliferation in OPCs derived from p7 mice or adult humans [17,115]. 

Differences between these experiments may be explained by OPC purity, experimental timing, or the age of animals from which cultures were derived. Such differences have broader implications for future studies of hormonal regulation of oligodendrogenesis. Firstly, an increasing number of studies demonstrate glia release factors that modulate neurogenesis [116,117,118]; hence, contamination of OPC cultures or the use of mixed glial cultures could influence oligodendrogenesis via indirect effects of IGF-1 on astrocytes or microglia, an important consideration for in vitro studies. Secondly, while co-applying BrdU and IGF-1 yields uptake of BrdU in OPCs, application of BrdU 24 h after IGF-1 treatment does not [17,113]. This suggests that IGF-1 may induce a small and/or transient increase in OPC proliferation. Indeed, in experiments with co-application of IGF-1 and BrdU, IGF-1-induced proliferation is inhibited by PI3K, MAPK kinase kinase 1 (MEK1), and Src-like tyrosine kinase inhibitors, which align with the mitogenic role of PI3K and MEK1 signaling in other cell types, as well as broader mitogenic properties of IGF-1 signaling [119,120]. Furthermore, the age at which primary cultures are obtained should be considered when comparing literature with cultured OPCs. OPC generation and migration through the developing rodent forebrain occurs in separate waves, with the final wave beginning around birth [121]. OPCs from the final wave compete with existing OL precursors and become the predominant OPC in many brain regions by p10. Although they maintain similar capacities for myelination, the responses of these separate OPC lineages to survival and proliferative factors are poorly understood. In fact, neonatal rat-derived OPCs may differ in their response to IGF-1, showing less IGF-1-induced differentiation to mature OLs as compared to their adult-derived counterparts [122]. This effect may be driven by differences in transcriptional profiles of proliferative and survival-related genes in neonatal- vs. adult-derived OPC cultures [122]. Lastly, culture conditions should be noted. Again, IGF-1 can act in concert with growth factors to promote the proliferation of OPCs, but IGF-1 alone may have little effect [123].

Altogether, although the primary effect of IGF-1 is to inhibit apoptosis [114], IGF-1 may induce a small and transient PI3K/MEK1-dependent proliferative effect on OPCs. In support of this, loss of IGF1R specifically in Olig1-expressing cells results in a small decrease in proliferating NG2+ cells in young (2-week-old) mice [111]. Additional experiments with, for example, live cell imaging on highly pure cultures to quantify cell cycle entry in real time would advance understanding of IGF-1’s effects on OPC proliferation. 

These studies demonstrate that IGF-1 stabilizes, and perhaps modestly amplifies, the OL progenitor pool. Further studies suggest that IGF-1 also promotes the commitment of glial progenitors to an OL fate. Indeed, early in vitro work posited that IGF-1 promotes the maturation of neonatal and embryonic rat-derived intermediate OPCs, as indicated by a higher percentage of O-2A progenitors progressing to immature OLs [112,124]. However, little else has been done to investigate whether and how IGF-1 promotes differentiation and maturation of OLs from OPCs beyond promoting cell survival. Similarly, few studies have addressed whether IGF-1 promotes the transcriptional or structural enhancement of myelinogenesis, either in development or during adulthood. While several studies demonstrate that IGF-1 upregulation increases myelin content in vitro and in vivo [124,125], this effect may be explained by greater numbers of surviving and differentiated OLs. One study has suggested that IGF-1 enhances transcription of myelin proteins from OLs [126]; however, this study utilized Northern blots in mouse mixed glial cultures. Studies with pure OL cultures and more quantitative methods of measuring transcription would strengthen our understanding of the effects of IGF-1 on OL maturation and myelinogenesis.

Ultimately, IGF-1 amplifies the number of mature, myelin-producing OLs in culture [100]. IGF-1 acts on multiple stages of OL development, from stem cell OL commitment to survival of mature OLs. This work in cell culture aligns well with in vivo studies in which IGF-1 signaling is either constitutively enhanced or reduced, leading to enhanced or reduced myelination, respectively. Specifically, mice deficient in GH or IGF-1 display widespread reductions in CNS myelination throughout development [127,128,129]. Conversely, mice overexpressing IGF-1 have larger brains with greater myelin content [130]. This is true as well when IGF-1 overexpression is restricted to astrocytes and OLs [131,132]. Loss of IGF1R specifically in either immature (Olig1+) or mature (PLP+) OLs results in developmental reductions in brain weight, OPC density, OL density, and myelination [111]. 

Each of these experiments utilized transgenic animals with constitutive transgene expression. Interestingly, while transgenic overexpression of IGF-1 produces a consistent elevation in brain weight and myelination throughout development, these measures stabilize by adulthood, suggesting that there may be a developmental window and/or diminishing effects for IGF-1 on OLs and myelinogenesis [125,132]. While studies in adulthood are limited, one study assessed OL turnover in a rat model of adult-onset loss of GH and IGF-1 production [133]. Following loss of GH/IGF-1 signaling, the total number of proliferating (BrdU+) cells in the corpus callosum decreased, as well as the number, but not the percentage, of BrdU/PDGFR-ɑ+ OPCs. Similar results were found for immature (glutathione-S-transferase-pi, GST-pi+ and adenomatous polyposis coli, APC+) OLs, suggesting a role for IGF-1 on OPC and OL survival in the adult brain [133]. Additional studies utilizing transgenic lines with temporally controlled genetic manipulation of IGF-1 signaling would greatly enhance our understanding of IGF-1’s role in oligodendrogenesis, specifically in adulthood. In addition, more detailed analyses of the mechanisms of IGF-1’s effects on OPC differentiation, OL maturation, and myelination would aid in determining whether IGF-1’s actions extend beyond survival and might enhance remyelination in disease contexts.

### 2.3. Insulin

Insulin is a 51 amino acid metabolic hormone that regulates glucose homeostasis by enhancing glycogen synthesis as well as the metabolism of other molecules such as lipids and certain amino acids [134]. In addition, insulin can promote cell division and growth, while also affecting behaviors such as food intake [135,136]. Insulin can be delivered to the CNS via circulation and transport across the BBB [137]. However, insulin transcription has also been detected in neural and glial cultures, suggesting that insulin can act in both an endocrine and paracrine fashion on CNS cells [138,139]. Insulin binds to the insulin receptor (IR), a cell surface receptor with tyrosine kinase activity that, in the CNS, is expressed in the olfactory bulbs, the arcuate nucleus of the hypothalamus, and the hippocampus [140]. The IR is also expressed in all types of CNS cells, including OLs [141,142]. In addition, insulin can bind to IGF1R, albeit with a lower affinity than IGF-1 [104].

#### Effects on Oligodendrogenesis

Given the evolutionary relatedness of insulin and IGF-1 and the known crosstalk between their receptors, it is not surprising that insulin exhibits effects on oligodendrogenesis that are similar to those of IGF-1. Indeed, insulin promotes adult rat NSC differentiation towards the OL lineage and promotes p6–8 rat OPC and OL survival in culture [107,108,143]. Furthermore, similar to IGF-1, insulin increases the percentage of differentiated OLs from cultured p6–8 rat OPCs, suggesting enhanced OPC differentiation and/or OL survival [143]. Notably, at the high concentrations (e.g., 5000 ng/mL) used in some of these experiments, insulin can bind IGF1R and act via the mechanisms detailed above. However, dose–response experiments suggest that insulin can also act independently of IGF1R at physiological concentrations [108]. In addition, insulin may have the ability to increase MBP levels in vitro. Specifically, neonatal rat OPCs prepared from mixed glial cultures show an insulin dose-dependent increase in MBP protein [144]. However, in this study, insulin had no direct effect on MBP mRNA levels, and similarly to IGF-1, the observed increase in MBP protein may be due to enhanced differentiation and/or survival of OLs [144]. The specific actions of IR on oligodendrogenesis remain poorly understood, and future studies should determine whether and how insulin modulates OL differentiation, survival, and maturation independent of IGF1R. For example, the effects of insulin could be tested in the absence of IGF1R signaling, either via genetic ablation or selective receptor antagonism of IGF1R. In addition, all of the work noted here was performed in vitro. Given the high insulin levels that accompany disorders such as adult-onset type 2 diabetes, future work should also seek to determine whether insulin, either through IR or IGF1R, or insulin resistance modulates adult oligodendrogenesis using in vivo models.

### 2.4. Prolactin

Prolactin is a 199 amino acid peptide that is best known for promoting lactation but also regulates diverse functions including sexual and parental behavior, immunomodulation, and osmoregulation [145]. Circulating prolactin is produced by the anterior pituitary and can cross the BBB; however, prolactin can also be produced locally by tissues such as the mammary glands, placenta, and brain (including regions such as the cortex, amygdala, thalamus, and hippocampus) [145,146,147,148,149]. Prolactin release by the anterior pituitary is environmentally modulated by a number of stimuli, including stress, daylength, and infant suckling, and is neurally modulated by a number of signaling molecules that exhibit stimulatory (e.g., thyrotropin releasing hormone, oxytocin) or inhibitory (e.g., dopamine, somatostatin) regulation of prolactin release [145,150,151]. 

The prolactin receptor (PRLR) is a transmembrane receptor of the type 1 cytokine superfamily [145]. PRLR activation induces a kinase cascade primarily involving the Janus kinase/signal transducer and activator of transcription signaling pathway, which contributes to cellular growth and proliferation [152]. PRLR can also act via PI3K and MAPK signaling pathways [145]. PRLR is expressed in several regions of the brain [153,154]; however, only one study to date has examined expression of PRLR in the OL lineage. Specifically, a subset of PDGFRα+ OPCs in the corpus callosum and spinal cord express PRLR [155]. This offers a potential mechanism by which prolactin may alter oligodendrogenesis in the CNS.

#### Effects on Oligodendrogenesis

Although the entirety of prolactin’s actions on the OL lineage are not known, prolactin may act on OPCs to enhance oligodendrogenesis. In NSC cultures derived from both the adult mouse subventricular zone and human embryos, prolactin stimulates NSC proliferation [156,157,158]. Furthermore, in OPC neurospheres (i.e., clusters of OPCs in culture) derived from the corpus callosum of adult female mice, prolactin treatment increases the number and size of OPC neurospheres and increases the proportion of OLs in culture, suggesting that prolactin enhances OPC proliferation and differentiation [155]. Interestingly, OPC proliferation, OL generation, MBP expression, and numbers of myelinated axons in the corpus callosum and spinal cord are all increased during pregnancy in mice, and heterozygous loss of PRLR function attenuates the pregnancy-associated increase in OPC proliferation [155]. Furthermore, administration of exogenous prolactin to virgin mice increases OPC proliferation [155]. Although this suggests that prolactin can act either directly or indirectly on OPCs to promote proliferation, these findings are in contrast to work performed in OPC-enriched neurosphere cultures derived from the adult rat hippocampus (with 75% A2B5+ cells), in which seven days of prolactin treatment had no effect on cell numbers and no effect on differentiation into MBP+ OLs [159]. Whether prolactin acts on OPCs to enhance proliferation merits further investigation. Interestingly, prolactin may exhibit some protective effects in demyelinating conditions [25,155]; however, beyond the few studies cited here, little else has been done to investigate whether and how prolactin acts on the various stages of the OL lineage outside of disease contexts. Our understanding of the effects of prolactin on oligodendrogenesis would benefit from work with highly pure OL cultures, targeted disruption of prolactin signaling in the OL lineage, and a closer investigation of the intracellular mechanisms that mediate prolactin’s direct and indirect effects on OPCs and OLs.

### 2.5. Melatonin

Melatonin is an indolamine neurohormone derived from the amino acid tryptophan by way of serotonin. It is produced primarily by the pineal gland, although small amounts of melatonin may also be produced by other regions of the brain [160]. In addition, retinal and gut tissue can produce melatonin for local action [161]. Melatonin production is indirectly controlled by the suprachiasmatic nucleus, and retinal exposure to bright light indirectly inhibits melatonin production [162,163].

Melatonin is released into circulation and readily crosses the BBB. Melatonin binds two G-protein coupled receptors, melatonin receptor 1 (MT1) and 2 (MT2). MT1 is coupled to G_i_ proteins that inhibit adenylyl cyclase cAMP production and, hence, protein kinase A (PKA) activity [164]. Additionally, MT1 is coupled to G_q/11_ proteins that stimulate phospholipase C (PLC) activity and MEK/ERK signaling. Melatonin binding to MT1 also activates Kir3 inward-rectifying potassium channels. MT2 activates similar cascades to MT1, but also inhibits guanylyl cyclase [164]. Through these receptors, melatonin entrains central and peripheral tissues to the circadian rhythm, thereby regulating sleep–wake cycles, circadian hormone release, metabolism, and other daily or seasonal rhythms. In addition, melatonin exerts anti-inflammatory actions by normalizing pro-inflammatory cytokine levels, inhibiting inflammatory signaling cascades, and scavenging free radicals [165,166]. One study has shown that neonatal rat OLs express, to some extent, both MT1 and MT2, suggesting that melatonin may regulate oligodendrogenesis [167].

#### Effects on Oligodendrogenesis

Interest in melatonin’s effects on oligodendrogenesis began with studies demonstrating that melatonin is neuroprotective against white matter damage [168,169]. However, the mechanistic effects of melatonin on oligodendrogenesis are largely unknown. In embryonic mouse-derived NSC cell culture, application of melatonin over five days increases the percentage of MBP+ cells as compared to vehicle or PDGF application [170]. This suggests that melatonin may enhance NSC-derived OL differentiation or survival. Similar increases in OL numbers were found with melatonin application to NSC neurospheres cultured from the adult mouse subventricular zone, suggesting that melatonin acts across development to enhance OL differentiation from NSCs [171]. 

Melatonin may also influence OL maturation. In studies of OL lineage cultures derived from neonatal rats, melatonin did not alter the number of immature (O4+) OLs but significantly increased the number of mature, myelinating (MBP+) OLs [167]. Moreover, similar effects were found in vivo. Specifically, neonatal rat pups subjected to uterine artery ligation to induce white matter damage were treated for three days with melatonin. While total (Olig2+) OL cell loss was not affected, mature (APC+) OL numbers were partially rescued in the cingulate and corpus callosum; this effect was thought to be through maturation because melatonin treatment did not affect OL cell death [167]. Together, these results suggest that melatonin does not alter OL differentiation but increases OL maturation. However, these results may be muddled by the fact that, in the in vitro experiments, the cultured cells included astrocytes and microglia, which also express the melatonin receptors [167,172]. Specifically, the authors demonstrated that melatonin attenuates microglial activation [167]. Hence, it is unclear whether melatonin acts directly on OLs themselves or whether it acts on surrounding microglia to promote OL maturation indirectly. Experiments with highly pure cultures of OLs or OPCs could address this question. 

Interestingly, one study more directly assessed the protective effects of melatonin on OL survival. Hypoxic conditions induce expression of caspase-3 in OLN-93 cells derived from p1 rats [173]. Applying melatonin to the culture medium attenuated hypoxia-induced caspase-3 expression, suggesting that melatonin acts on OLs to inhibit apoptotic cascades. 

Overall, melatonin may regulate oligodendrogenesis by promoting NSC commitment to the OL lineage, OL maturation, and OL survival. However, the number of studies supporting these claims are small, and several questions remain regarding both the physiological and mechanistic effects of melatonin on oligodendrogenesis. For instance, the effects of melatonin on OPC proliferation have not been tested. Given MT1 and MT2’s known interactions with MEK/ERK proteins, future studies could, for example, test the mitogenic role of melatonin on OPCs and/or the role of MEK signaling in melatonin’s regulation of oligodendrogenesis. Furthermore, the effects of melatonin on OL survival have only been tested under hypoxic condition; whether and how melatonin promotes OL survival under baseline conditions is not known. In addition, given the expression of melatonin receptors on microglia and astrocytes, it remains unclear whether results from existing in vitro experiments are due to direct actions of melatonin on OLs or indirect actions on contaminating glial cells. Additional studies with melatonin receptor signaling disrupted specifically in the OL lineage would be interesting and beneficial.

## 3. Steroid Hormones

Steroid hormones are hydrophobic molecules synthesized from cholesterol that consist of three cyclohexanes and one cyclopentane with alternating enol and ketone groups [174]. This class of hormones encompasses a wide range of molecules with differing functions, including the regulation of reproduction, stress, and metabolism. As lipophilic molecules, steroids bind to carrier proteins in the bloodstream, such as corticosteroid-binding globulin for the glucocorticoids [175], but readily cross the plasma membrane. Once inside the cell, steroids bind to intracellular receptors, which may be sequestered in either the cytoplasm or nucleus. Steroid hormone binding at the ligand-binding domain of the receptor induces translocation of cytoplasmic receptors to the nucleus. Broadly, ligand-bound intracellular receptors act as transcription factors. Specifically, the DNA-binding domain of the receptor recognizes distinct sequences in the promoter regions of genes, for example the glucocorticoid response element or estrogen response element. Binding of the steroid–receptor complex to these elements ultimately alters gene expression. In contrast to this genomic route of action, steroids can also bind specific membrane-bound receptors. These receptors act through fast-acting second messenger systems and allow for rapid induction of kinase cascades with various cellular functions. 

For this review, we will focus on a subset of steroid hormones synthesized primarily in the adrenal cortex and gonads, namely the stress and sex hormones. Stress hormones such as glucocorticoids, and sex hormones such as estradiol, progesterone, and testosterone, impact neurogenesis and gliogenesis in the CNS, as well as OL survival and remyelination in multiple sclerosis and other myelin-related diseases [25,31,176,177]. As we will discuss below, steroid hormones influence OL development and myelination in both development and adulthood. In particular, cells across the OL lineage express the classical nuclear receptors of each steroid hormone, as well as additional membrane receptors. Through both genomic and non-genomic mechanisms, then, steroids act to increase OPC differentiation and enhance maturation/myelination of OLs.

### 3.1. Glucocorticoids

Glucocorticoids (GCs) are one of the primary stress hormones for almost all animals. This family includes endogenous cortisol (the primary GC for humans) and corticosterone (Cort; the primary GC for rodents), as well as synthetic hormones such as dexamethasone (Dex). GCs are released in a circadian manner, in response to physiological cues, and under stressful conditions [178]. They are the end product of the hypothalamic–pituitary–adrenal axis, which starts in the hypothalamus and ends with the release of GCs from the adrenal cortex into the bloodstream [179,180]. 

GCs can bind to two receptors: mineralocorticoid receptors and glucocorticoid receptors (GRs), both of which are intracellular receptors typically located in the cytoplasm [181]. The activation of these receptors produces effects across the body, leading to the mobilization of energy substrates and the suppression of inflammation, among other functions [178]. GRs are also found in all cell types in the CNS [178]. In particular, GRs, and to a lesser degree mineralocorticoid receptors, have been identified in both immature and mature OLs [142,182,183,184]. The identification of GRs on OLs provides a direct mechanism by which stress hormones may alter OL development and myelination. Here, we describe GC effects on developmental oligodendrogenesis separately from effects in adulthood because, unlike the other hormones, there is a large amount of literature specifically on GC’s impact in adult-derived cells and adult animals.

#### 3.1.1. Effects on Developmental Oligodendrogenesis

GCs play an important and complex role in early OL cell development: while GCs can increase OL differentiation and maturation, their effects may depend on both timing and dosage. Several seminal studies found that postnatal adrenalectomy profoundly changes myelination in the developing brain [185,186], providing an initial indication that GCs may be involved in OL development and myelination. Some of the first evidence from in vitro studies suggested that GCs promote differentiation and survival of cells along the OL lineage. In mixed glial cell cultures generated from one-week-old rat pups, hydrocortisone not only enhanced survival of all glial cells, it also increased the ratio of OLs relative to other glial cell types [187]. In subsequent studies, an increase in the number of OPCs was also observed following Cort or Dex application to OPC-enriched cultures (90% A2B5+), and GCs protected against inflammatory cytokine-induced cell death [188]. However, administering Dex in vivo to neonatal rats for five days reduced the number of OPC (O4+) cells in the corpus callosum, and induced morphological changes associated with cell death [189]. Thus, GC effects on survival may differ in vivo or when levels of GCs exceed the physiological range.

GCs act to regulate the timing of OPC differentiation into OLs. In cultured OPCs purified from p8 rat brains, when in the presence of mitogens, GCs induced slowing of proliferation and increased OL differentiation from OPCs [44]. However, such stimulatory effects of GCs on OL differentiation are not always observed. For example, in OLN-93, an oligodendroglial cell line derived from p1 rats, Dex inhibited the expression of CNPase, a marker of OL differentiation [190]. Differences in GC effects on differentiation may be dependent on developmental stage. In a study with cells cultured from embryonic rats, 15 days of Dex treatment increased the OL markers CNPase and MBP, while 25 days of Dex treatment inhibited these same markers [191]. It is unclear, however, whether this reduction in OL markers was due to the prolonged exposure to Dex or whether this effect was dependent on cellular developmental age. Furthermore, this study utilized aggregated cell cultures containing both neurons and glia; thus, interactions between cell types cannot be ruled out.

GCs may also affect OL maturation and myelogenesis in early development. Hydrocortisone treatment of primary cell cultures derived from newborn rat cortices increased transcripts and protein for three myelin markers: glycerol phosphate dehydrogenase (GPDH), a general marker of OLs, as well as MBP and PLP, proteins associated with mature OLs and the myelin sheath [192,193]. These effects of hydrocortisone were only observed when treating cells that had been in culture for at least nine days [193]. Future studies should therefore test whether these effects are age-dependent by directly comparing OL cultures derived from neonatal animals with cultures derived from animals later in development. 

Importantly, many of the studies described above used mixed glial cultures, and therefore, observed effects on oligodendrogenesis could occur indirectly via GC-induced alterations in astrocytes or microglia. Indeed, one study treated purified OPC and OL cultures derived from neonatal rats with Dex and found that Dex had no effect on oligodendrogenesis and altered few gene transcripts [194]. In contrast, Dex led to widespread transcriptional changes in microglia and astrocyte cultures [194]. Thus, culture purity is an important consideration for future in vitro studies, and it remains unclear how interactions with other glial cells might affect oligodendrogenesis.

#### 3.1.2. Effects on Adult Oligodendrogenesis

GCs can also have an effect on OL differentiation, proliferation, and maturation in the adult brain. In line with observations in the developing brain, GCs inhibit OPC proliferation in adults. For example, in adult adrenalectomized rats, prolonged GC exposure (15 days of 10 mg/kg) inhibits OPC proliferation, leading to fewer NG2+ cells in white and grey matter across the forebrain, including in much of the hippocampus [195]. A second study also found that one week of daily Cort injections (40 mg/kg) led to reduced proliferation of BrdU+/NG2+ OPCs in the adult rat hippocampus in the molecular layer and hilus regions, but not in the granule cell layer [196]. Furthermore, 15 days of chronic unpredictable stress decreased the number of BrdU+ OPCs across the cerebral cortex of the adult rat [197]; this effect only appeared three weeks following stress exposure, and while overall numbers were reduced, the percentage of BrdU+/NG2+ cells was not changed [197]. It is worth noting that results from these studies are unable to determine whether stress and GCs have an effect on OPC survival or whether reduced numbers are due to increased OPC differentiation into mature OLs.

While these studies identified fewer OPCs in the hippocampus following GC administration, others have found that stress exposure increases OL markers such as GPDH in the adult hippocampus [198,199]. GPDH is an enzyme that is unique to OLs in the rodent brain and is known to be upregulated by GCs [200,201]. A study by our own lab found that seven days of either immobilization stress or Cort injections increased oligodendrogenesis in the dentate gyrus of the adult rat hippocampus [21]. Specifically, stress and Cort decreased neurogenesis and increased oligodendrogenesis, indicated by a greater percentage of BrdU+ cells co-labeled with MBP. In addition, in a tamoxifen-inducible Nestin–CreER transgenic mouse line with NSCs fluorescently identifiable by yellow fluorescent protein (YFP), we found that Cort induces oligodendrogenesis in the hippocampus, with a greater percentage of YFP+ cells co-labeled with GST-pi, a marker of immature to mature OLs. Furthermore, exposure of cultured NSCs to Cort increased the pro-OL transcription factors Olig1 and Olig2 and the percentage of MBP+ cells. These effects were found to be dependent on GR signaling; blocking GRs with a dominant negative viral vector led to lower numbers of OLs and reduced pro-OL factors compared to controls [21]. 

Taken together, a complex picture emerges for GC effects, including decreases in OPC proliferation and increases in OLs. These reported studies utilized different stress timelines and analyzed different markers along the OL lineage. It is plausible that stress exposure, and/or stress hormones such as Cort, may reduce the number of dividing OPCs and instead push OPCs to differentiate into immature or mature OLs. Future studies should examine OPCs and OLs within the same study to test this hypothesis. 

GCs also have an impact on adult OL maturation and myelin morphology. In a study by Miyata et al. (2011), daily water immersion and restraint stress for three weeks increased plasma Cort levels and induced morphological changes in OLs in the corpus callosum, resulting in greater OL arborization compared to control animals. This was replicated in vitro via administration of Dex for two days in OL cell cultures. These stress-induced morphological changes are dependent on serum glucocorticoid-regulated kinase 1 (SGK1) and endogenous N-myc downstream-regulated gene 1 (NDRG1) signaling. Stress exposure leads to increased SGK1 phosphorylation, and subsequent increases in NDRG1 phosphorylation; together, SGK1 and NDRG1 upregulate the expression of adhesion molecules in OLs, specifically N-cadherin, and alpha- and beta-catenin, molecules involved in the stabilization of adherent junctions. While many other adhesion molecules expressed in OLs have been shown to aid in OL–axon signaling, promoting myelination [202], the functional role for these particular adhesion molecules in OLs remains unknown. In OL cultures derived from neonatal rats, overexpression of SGK1 and NDRG1 was sufficient to replicate the effects of Dex and stress, confirming a role for this pathway in stress-induced alterations of OL morphology [203]. A subsequent study found that exposure to acute stress also led to increased SGK1 expression in mature, MBP+ OLs. This effect was absent in adrenalectomized mice and restored with Cort injections, indicating that Cort is necessary for this effect [204]. Cort-induced changes in SGK1 expression were observed in white matter OLs, but not in grey matter OLs [204]. Future work could aim to determine why only grey matter OLs were affected.

### 3.2. Sex Hormones

Sex hormones, including estrogens, progestogens, and androgens, all modulate oligodendrogenesis and myelogenesis [31,205]. Interestingly, males and females display regional differences in white matter density [206,207]. Sex hormones might account for some of these sex-specific patterns of myelination.

#### 3.2.1. Estrogens

Estrogens, the major family of female sex hormones, are produced primarily by the ovaries. Estrogens have many physiological functions for both male and female animals, including the promotion of sexual maturation. Estrogen receptors (ERs) are found in many different cell types, including in OLs [208,209,210,211]. There are three major classes of endogenous estrogens: estrone, estradiol, and estriol. Of these, 17-β estradiol (E2) is considered to be the most potent and the most prevalent [212]. Interestingly, however, an optical isomer of E2, 17-α estradiol, is found at higher levels in the brain and can be produced in both sexes following gonadectomy [213]. While produced at higher levels in females, males also produce estradiol [214,215,216]. Specifically, tissues that contain aromatase, including in extragonadal sites, convert testosterone to E2 [217,218,219]. 

Estrogens primarily act at two intracellular receptors, ERα and ERβ, both members of the nuclear receptor family. While both isomers of estradiol are able to bind to these ERs, ERα and ERβ differ in their localization in both the body and within the CNS [220,221]. In addition to intracellular ERs, estrogens can activate membrane-bound receptors such as the G-protein coupled receptor, GPR30, which produce more rapid physiological responses [222,223,224]. Binding of GPR30 is estrogen selective; other hormones, including progesterone, cortisol, and testosterone, are not able to bind to GPR30 [225,226]. Collectively, activation of these receptors leads to the many downstream effects of estrogens. 

Importantly, NSCs, OPCs, and mature OLs express all three forms of estrogen receptors: ERα, ERβ, and GPR30 [208,209,210,211,227,228,229,230]. Interestingly, in addition to being found in the nuclei, ERα and ERβ can also be localized in the membrane and cytosol of OLs, and the relative expression and localization of these receptors may change along the OL lineage [208,211,228]. For example, NSCs display higher expression of ERβ relative to ERα [230,231]. Localization of ERs may also differ based on OL maturity. For example, one group identified ERα localized primarily in the nucleus and ERβ primarily in the cytoplasm of OLs [211]. In contrast, others showed ERα expression in the cell membrane and perikaryon in addition to the nucleus, while ERβ was located mainly in the nucleus and only to a lesser extent along the membrane [209]. These discrepancies may arise from differences in the age or maturity of the cultured cells. For example, while OL cultures express both ERα and ERβ in the cytosol and nucleus, nuclear compartmentalization of both ERs increases as cells mature [228]. Interestingly, the relative density of ERs in OLs also differs based on sex. Levels of ERα in mature OLs are eight-fold higher in females than males [228], indicating that estrogens may differentially affect males and females in part due to differences in ER expression. Regardless of localization or relative expression, activation of these receptors leads to changes in differentiation, proliferation, and maturation across OL development.

#### Effects on Oligodendrogenesis

Broadly, estrogens, and in particular, estradiol, regulate proliferation and differentiation across the OL lineage, beginning with NSCs. Specifically, E2 promotes embryonic rat NSC proliferation and differentiation in vitro. Notably, E2-induced proliferation is dependent on the activation of nuclear ERs, while E2-induced differentiation is dependent on membrane-associated ERs [232]. Estradiol’s effects on NSCs may also depend on cell culture conditions and interactions with other factors. For example, E2 prompts stem cells to differentiate into OL progenitors only when there are low levels of mitogens or other differentiation factors [230]. Under conditions where mitogens are present, and when there is physiologically appropriate dosing of E2, NSC differentiation is instead biased towards neuronal, rather than glial, cell fates [227]. Age is also a factor, as E2 only increases the ratio of neurons to glia in embryonic NSCs, not in adult NSCs [231]. 

While the above studies did not explicitly test the relative contributions of ERα and ERβ in E2-mediated effects, others have focused on the specific roles of particular ER receptors. ERβ ligands inhibit proliferation of mouse embryonic stem cells, and ERβ knock-out mice display enhanced oligodendrogenesis [233], suggesting that activation of ERβ in particular may promote stem cell differentiation into neurons and prevent precocious oligodendrogenesis. Little is known about the specific effects of ERα binding on NSC development. Indeed, activation of these different receptors may lead to diverging effects on oligodendrogenesis and requires future investigation. 

Like their neural stem cell counterparts, OPCs are also affected by estradiol. In vitro, E2 delays the exit of OPCs from the cell cycle in a dose-dependent manner in response to mitogen withdrawal [229]. This allows OPCs to undergo additional rounds of cell division, and ultimately, can be interpreted as an estradiol-induced increase in OPC proliferation. In the presence of mitogens, however, E2 does not affect OPC proliferation [228]. In addition to effects on proliferation, E2 can also enhance rodent OPC differentiation and maturation, leading to thicker branching in the subsequent OLs [229]. In one study, tamoxifen, an ERα selective agonist, mimicked the E2 effect on proliferation, but not its effect on branching [229]. This indicates that estradiol-induced changes in OPC proliferation might be mediated by Erα, while changes in cell morphology and maturation are instead mediated by ERβ. Interestingly, though, in a separate study, tamoxifen promoted OPC differentiation into OLs, stimulating progenitors to become mature OLs, suggesting that ERα may indeed play a role in OPC differentiation. This effect was abolished in the presence of a pan-ER antagonist [234]. Similarly, diosgenin, a steroid precursor, promoted OPC differentiation into mature OLs through an ER-dependent mechanism; differentiation was blocked by a pan-ER antagonist, but not by either GC or progesterone receptor antagonists [235]. Estradiol-induced increases in OPC differentiation may also occur through more rapid activation of a PI3K/AKT/mammalian target of rapamycin signaling pathway [236]. This pathway has been shown to be required for OPC differentiation into immature OLs [237,238]. Overall, through both genomic and nongenomic mechanisms, estrogens appear to stimulate OPC differentiation.

Similar to findings in OPCs, estrogens can also stimulate differentiation and maturation of immature and mature OLs, respectively [211,239,240]. In vitro, incubation of OLs with E2 increases cell branching and increases the number of cells with a snowflake shape, a stage in OL development that typically precedes formation of myelin sheaths [211]. In mature and myelinating OLs, estradiol also mediates remodeling of the cytoskeleton, acting via membrane-bound ERs. Notably, E2 and 17-α estradiol have opposing effects; while E2 induces a loss in microtubules and inactivates actin filaments, 17-α estradiol increases the percentage of cells with actin filaments, indicating stabilization of the cytoskeleton [240]. This remodeling in mature OLs is important for functions such as OL extension and axon wrapping, which is relevant not only in a developmental context, but also across the lifespan. Future work should aim to explore these different pathways and how activation of different membrane ERs and nuclear ERs lead to changes in OL maturation.

#### 3.2.2. Progestogens

The steroid hormone progesterone is part of a larger family of progestogens and is an important intermediate for other neurosteroids, including androgens and corticosteroids [241,242,243]. Although progesterone is commonly known for its role in the maintenance of pregnancy, it also has a wide range of functions in the body and throughout the CNS, including effects on sleep regulation and immune function [243]. For females, the corpus luteum in the ovaries is the major site of progesterone production. However, progesterone is also produced in the adrenal glands, in the placenta during pregnancy, and importantly, in the CNS of both females and males after birth and into adulthood [244]. Like all steroid hormones, progesterone is synthesized from cholesterol, which is then converted to the progesterone precursor, pregnenolone. The enzyme 3-β hydroxysteroid dehydrogenase (HSD) converts pregnenolone into its primary form, progesterone. Progesterone can also be metabolized by 5-α reductase to dihydroprogesterone, which is then metabolized further by 3-α HSD to allopregnanolone [245]. These metabolites have additional functions throughout the CNS, as will be described below. 

As with other steroid hormones, progesterone acts on two primary receptor classes: classical nuclear receptors and membrane-associated receptors. There are two forms of nuclear progesterone receptors (PRs): PRα and PRβ. Anatomically, these receptors are localized throughout the brain and spinal cord [246]. Functionally, PRβ is a more potent transcriptional activator, while PRα is primarily a transcriptional repressor [247,248]. Progesterone can also act on two forms of membrane-associated receptors to drive rapid, non-genomic effects: a seven transmembrane domain membrane progesterone receptor (mPR) and membrane-associated progesterone receptor membrane component 1 (PGRMC1), which was previously referred to as 25-Dx [246]. Together, progesterone acts at these receptors to produce downstream actions in both the periphery and in the CNS.

Nuclear PRs have been identified in glial cells, and in particular, in OLs in both the brain and spinal cord [239,249,250]. Interestingly, PR expression increases with the addition of estradiol to primary glial cell cultures, suggesting that PR expression can be regulated by other sex hormones [239,249]. Membrane-bound PRs have also been identified in the spinal cord [251]. In the brain, however, mPRs are typically only found in neurons and are only expressed in OLs following injury, suggesting a selective role for mPRs in injury recovery [252]. Few have described how PR expression changes across the OL lineage. This will be an interesting area for further research and will provide insight into the mechanisms involved in progesterone’s effects on OLs. 

Interestingly, OLs not only express PRs, but they also directly synthesize progesterone and its precursor, pregnenolone. Synthesis of pregnenolone was first observed in glial cultures containing 60% OLs and occurred at the same time as OL differentiation [253]. Enzymes for progesterone synthesis have since been detected in OL cultures, and changes in expression of these enzymes coincide with the differentiation of bipotential O-2A cells [254]. Later work confirmed that OPCs, and to a lesser extent mature OLs, express mRNA of 3-β HSD, the key steroidogenic enzyme that converts pregnenolone to progesterone [255,256]. OLs and their precursors also contain enzymes for progesterone metabolism [256,257,258]. In contrast to 3-β HSD, the metabolic enzyme 5-α reductase is expressed five-fold higher in mature OLs relative to progenitors [256]. The enzyme 3-α HSD, which converts 5-α dihydroprogesterone to allopregnanolone, has also been observed in early progenitor cells, indicating a dissociation in the timing and pathways of progesterone metabolism in the OL lineage. The expression of PRs and the synthesis of progesterone and its metabolites across the OL lineage suggest that OLs may respond to progestogens in both an endocrine and autocrine fashion to regulate oligodendrogenesis. 

#### Effects on Oligodendrogenesis

In general, progesterone and its metabolites stimulate OL proliferation, differentiation, and maturation across all stages of development [259]. At the NSC and OPP stage (PSA-NCAM+), progesterone stimulates cell proliferation. This effect is primarily mediated by conversion of progesterone to its metabolite, allopregnanolone, as blocking this enzymatic conversion inhibits progesterone’s effects [257]. Allopregnanolone has also been shown to directly induce proliferation in both human and rat NSC cultures isolated from early in development [257,260,261]. These mitogenic effects are mediated by allopregnanolone acting as a positive allosteric modulator of GABA-A receptors; this in turn activates voltage-gated L-type calcium channels and drives cAMP response element-binding protein signaling [257,260,261]. While allopregnanolone largely stimulates progenitor proliferation, its effects follow a bell-shaped curve, with high levels inhibiting proliferation [257,261]. Thus, use of progesterone and its metabolites as a neurogenic agent should take into account hormone concentrations.

In OPCs derived from rodents, progesterone enhances proliferation and differentiation in vitro, increasing their overall number and prompting OPCs to branch and mature into OLs [229,256,257,259,262,263,264]. Unlike findings in OPPs, progesterone’s effects on OPC proliferation were not mimicked by the metabolite allopregnanolone, even at high concentrations [264]. Mechanistically, progesterone’s effects on OPC proliferation and differentiation are mediated through PR signaling, as PR antagonists block these effects [264,265]. One study took these findings further and identified that, while mouse embryonic OPCs express both PRα and PRβ, signaling through the PRβ receptor mediates progesterone’s stimulatory effects on OPC proliferation and differentiation [265]. Activation of PRβ leads to the upregulation of oligodendroglial genes ranging across the OL lineage, such as NG2, MBP, and CNPase. Indeed, an mPR-specific agonist did not alter OPC proliferation or differentiation, confirming that progesterone’s effects on oligodendrogenesis during development occur via a genomic mechanism [265]. 

Progesterone stimulates the differentiation of OPCs; therefore, it is no surprise that adding progesterone to cultures derived from rodents increases the number of immature and mature OLs [236,249,253]. At the immature OL stage, progesterone promotes MBP expression, presumably indicating increased differentiation into mature, myelinating OLs [265], but does not increase pre-OL proliferation, as progesterone does not alter the incorporation of BrdU into A007 or O4+ OLs [229,236]. Interestingly, the same dose of progesterone increases immature OL numbers to a greater extent in OLs cultured from 2–3-day-old female mice compared to males, suggesting the magnitude of progesterone’s effects depends on sex [236]. In addition to increasing numbers of immature OLs, progesterone also increases the number of mature, myelinating OLs (MBP+/CNPase+ immunoreactive cells), MBP and CNPase mRNA, and myelin protein expression [67,190,239,254,266,267,268]. Increases in MBP protein may be due to increased numbers of mature OLs, although there is some evidence to suggest that progesterone not only increases MBP+ cell numbers, but also MBP fluorescence intensity within a single mature OL in vitro [267]. The PR again is implicated mechanistically, especially for progesterone-induced increases in MBP [263,267]. In one study using cell cultures derived from newborn rats, selective antagonism of the PR did not alter progesterone-induced increases in the number of MBP+ cells; however, it did reduce overall MBP fluorescence intensity [267]. Furthermore, while progesterone agonists increased MBP expression, cell cultures from PR knock-out mice treated with progesterone no longer showed increases in MBP levels [263]. Future studies could aim to untangle whether progesterone-induced increases in MBP and CNPase represent higher numbers of myelinating OLs or greater myelination by existing OLs.

#### 3.2.3. Androgens

Androgens are a class of steroid hormones derived from cholesterol by way of the progestogens. The androgen dehydroepiandrosterone (DHEA) is the primary androgen/estrogen precursor and is the least potent androgen [269]. DHEA can be converted to androstenedione (A4) or androstenediol (A5), both of which have weak to moderate androgen activity. Both A4 and A5 can be converted to testosterone, the primary circulating androgen in males [269]. Testosterone can be further metabolized by 5-α-reductase to the most potent androgen, dihydrotestosterone (DHT), locally in tissues such as the genitalia, skin, prostate gland, liver, and brain [269]. Interestingly, isolated myelin sheaths also display robust 5-α-reductase activity [270,271]. Notably, androgens are the precursors to estrogens. Via the enzyme aromatase, A4 is converted to estrone, while testosterone is converted to estradiol. An important consideration in the study of androgens, therefore, is whether observed effects of androgen administration are due to direct action of androgens or indirect action via conversion to estrogens. Many studies attempt to address this by administering DHT, which cannot be aromatized. Notably, however, DHT metabolites have been shown to bind ERβ, which is an important caveat for all experiments utilizing DHT [272,273].

Although circulating levels of androgens are higher in males, both males and females produce androgens [274]. The zona reticularis of the adrenal cortex primarily produces the weak androgens DHEA, A4, and A5, while the gonads are the primary source of testosterone. In males, androgen production begins early in development with a fetal surge of testosterone which (primarily via conversion to DHT or estradiol) masculinizes the genitalia, brain, and other organs of the developing male [275]. Androgen levels then remain relatively low until puberty [274]. In females, androgen levels remain low until puberty, and the absence of androgens is a primary factor determining feminization of the genitalia, brain, etc. [275,276]. 

Androgens primarily act on the androgen receptor (AR), an intracellular receptor sequestered in the cytoplasm [277]. In addition, evidence suggests that androgens can act via membrane-bound receptors to activate rapid second-messenger systems, including the PI3K/AKT and MAPK/ERK pathways [278,279]. The AR is expressed throughout the brain; however, very few studies have addressed whether androgens act directly on OLs via the AR or indirectly via actions on surrounding cell types. In rodents, one study examined the brains of rats ranging from embryonic day 20 to p86 and found no AR immunoreactivity in mature galactosylceramidase (GalC+) OLs at any age [280]. In contrast, one study of the prefrontal cortex of adult male and female rhesus macaque brains revealed that, while the majority of AR-expressing cells were astrocytes, roughly 5% of CNPase+ OLs colocalized with AR [281]. This species difference is reflected in transcriptomic analyses which suggest that AR expression is essentially undetectable in mouse OLs of any stage, while human OLs express AR to a low degree [142]. In addition, no studies have examined whether OLs express fast-acting, membrane-bound ARs.

#### Effects on Oligodendrogenesis

Despite the low-to-absent expression of AR in OLs, several studies have indicated that the manipulation of androgens has profound effects on OLs and myelin. Broadly, male rodents have greater OL cell density in white matter regions such as the corpus callosum, fornix, and spinal cord [206,282], although females have greater overall glial proliferation and cell death [206]. These sex differences in rodents arise as early as p5 and continue into adulthood [282]. Specific manipulation of androgens reveals that these sex differences occur, at least in part, due to the AR and not simply via conversion to estrogens. For example, AR inhibition in male mice or DHT administration to female mice from p0 to p10 reverses the sex differences in corpus callosum OL density, and constitutive genetic deletion of AR in the CNS of males feminizes OL density and MBP expression throughout development and adulthood [282]. In humans, estimates of white matter volume correlate strongly with bioavailable testosterone in male adolescents [283]. Moreover, this relationship is stronger in males with a polymorphism in the AR gene which is associated with greater androgen-dependent gene expression. Together, these studies suggest a direct role for the AR in promoting OL and myelin density in white matter tracts of male rodents and humans.

The mechanism by which androgens and ARs alter oligodendrogenesis to bring about these sex differences remains somewhat unclear. Rat and human NSCs express the AR, and human embryonic NSCs transcriptionally respond to DHT in vitro [284,285,286]. Although some conflicting evidence exists, androgens may increase the proliferation of cultured NSCs [284,285,286]. Early work also suggested that sex hormones alter glial proliferation and/or survival. Specifically, gonadectomy in adult male mice both decreases corpus callosum OL cell density and increases the number of BrdU+ cells, suggesting a role for sex hormones in glial proliferation and/or cell death [206]. However, the application of testosterone to neonatal rat-derived OPCs in culture does not alter BrdU incorporation, arguing against a direct role for androgens in OL proliferation [229].

Interestingly, androgens may promote OL cell death. Treating neonatal rat OLs in culture with testosterone induces a small amount of OL cell death and potentiates excitotoxicity induced by exposure to α-amino-3-hydroxy-5-methyl-4-isoxazolepropionic acid (AMPA) and kainate [287]. This potentiation of excitotoxicity can be blocked by an AR antagonist, but not by aromatase inhibitors, suggesting that this effect is dependent on androgens and AR. The exact mechanism is unclear; while testosterone potentiates AMPA and kainate receptor-induced calcium influx, testosterone does not appear to alter the expression of glutamate receptor subunits in cultured OLs [287]. In line with this, exposure of cultured neonatal mouse OLs to DHT decreases phosphorylated AKT expression and increases the number of OLs expressing caspase-3, suggesting that androgens can induce OL cell death [236].

In summary, there are many questions remaining in regard to androgens’ role in oligodendrogenesis. For example, it remains unclear whether OPCs or OLs express cytoplasmic and/or membrane-bound ARs. Furthermore, multiple lines of evidence suggest that there is an AR-dependent sex difference in OL density in vivo; however, the underlying mechanism of this sex difference remains unresolved, as in vitro work suggests that OPC proliferation is not altered by androgens. Such questions can be definitively addressed with future well-controlled in vitro studies and transgenic models with AR manipulations targeted to the OL lineage.

## 4. Non-Classical Hormones: Neurohormones, Neuromodulators, and Neurotransmitters

This review has focused on “classic” endocrine hormones; however, many hormones (including some discussed here) are produced in the CNS and can act in a paracrine fashion in the brain via synaptic or extrasynaptic transmission. Future research could investigate how neuropeptides that are not deemed to be classic hormones, such as corticotropin releasing hormone (CRH), the neurotransmitter norepinephrine (NE), and secretin hormones such as vasoactive intestinal peptide (VIP) and pituitary adenylate cyclase activating peptide (PACAP), affect oligodendrogenesis. 

Existing literature on CRH is limited and only indirectly applies to oligodendrogenesis [288,289], although CRH elevates cAMP levels in OPCs [290]. Existing research regarding NE is more abundant, and adrenoreceptors are found across the OL lineage. Specifically, the α1-adrenergic receptor is prevalent in both OPCs and differentiated OLs [142,291,292], and β1-adrenergic receptor expression has been detected in mouse OPCs and rat GalC+ OLs [142,293]. NE induces α1-adrenergic receptor-dependent second messenger signaling in neonatal rat OL cultures [291]. However, in rat embryonic day 20 OPC cultures, signaling through α1 receptors does not affect OPC proliferation [294], and the effects of these receptors on oligodendrogenesis remain unresolved. In contrast, activation of β-adrenergic receptors inhibits proliferation and induces the differentiation of cultured OPCs [294]. Future research should continue to investigate how catecholamines, CRH, and other neuromodulators affect oligodendrogenesis throughout the postnatal and adult period and if effects are observed following injury. 

Interestingly, there is a growing body of literature suggesting that peptides VIP and PACAP may also influence oligodendrogenesis. These homologous proteins are members of the secretin superfamily and have been increasingly implicated in a diverse set of functions in the body, such as regulation of circadian rhythms, smooth muscle tone, immune function, and cell proliferation [295,296]. Both VIP and PACAP are expressed in many regions of the brain [297]. VIP and PACAP bind to the VIP/PACAP receptors, VPAC1 and VPAC2; in addition, PACAP binds an additional receptor, PAC1, with high affinity [296]. While no studies have demonstrated protein-level expression of VPAC1 or VPAC2 in OPCs or OLs, RNA transcriptomic analyses suggest that VPAC2 is enriched in mouse OPCs, but not mature OLs [142]. In addition, PAC1 mRNA and PAC1 protein expression have been detected in immature to mature rat OLs in vitro and in vivo [298,299]. Thus, VIP and/or PACAP may influence the OL lineage. Consistent with this, early and intermediate neonatal rat OPCs respond to VIP and to PACAP by elevating cAMP levels in vitro [290]. While the specific actions of these peptides on oligodendrogenesis are largely unknown, evidence suggests that PACAP increases neonatal rat OPC proliferation in vitro [299]. PACAP may also delay myelination both in vitro and in vivo [299,300]. Given that the expression of receptors for VIP and PACAP change over the course of the OL lineage, additional research with selective agonists and antagonists for each receptor would greatly aid in our understanding of the role of these peptides in the various stages of oligodendrogenesis.

## 5. Future Directions

In this review, we have discussed the roles of numerous hormones in the regulation of oligodendrogenesis; however, there are many avenues for future work in this field. For example, much of the work we have described has built upon investigations in vitro that utilize classic, but limited, pharmacological approaches and raw counts of cells from discrete, but somewhat arbitrary, time points. In addition, many of the studies we described focused on only a particular timepoint in the OL lineage, often through the use of just one cellular marker. Future studies will require careful examination across the OL lineage, utilizing multiple markers of OL staging and ideally looking at markers for proliferation (such as BrdU) and survival (such as caspase-3) all within the same study. This will enable optimal interpretation of a hormone’s effects on OLs, and will allow us to determine whether changes in numbers of OPCs or mature OLs are due to proliferation, differentiation, or survival. 

Furthermore, for most of the hormones discussed, there remain substantial gaps in our understanding of the fundamental mechanisms governing the intracellular response to the hormone and the subsequent fate of the OL lineage cell. These gaps could be addressed with carefully controlled in vitro experiments. In particular, the field is ripe for studies that utilize modern techniques for targeted manipulation of hormones and their receptors and precise measurement of proliferation, differentiation, and myelinogenesis to dissect the role of these hormones on OPCs, OLs, or surrounding neurons, astrocytes, and microglia.

In addition to direct effects on the OL lineage, hormones may indirectly affect oligodendrogenesis through their interactions with other hormones. For example, high levels of GCs reduce thyroid functioning, leading to less conversion of T4 into the active T3; additionally, thyroid hormones, as previously described, also tend to have pro-oligodendrogenesis effects [301,302]. Thus, high levels of GCs could in fact inhibit oligodendrogenesis through indirect interactions with the thyroid system. T3-induced OPC differentiation could also be enhanced through interactions with other OL differentiation factors such as IGF-1, among others [44,303,304]. TH levels are positively associated with IGF-1 levels [303,305], and IGF-1 is upregulated following TH exposure in adult rat brains. Progesterone also interacts with the IGF-1 system. Specifically, progesterone upregulates IGF-1 and the IGF binding protein 6 (IGFBP-6) in OPCs [190]. IGF-1, as previously noted, broadly increases oligodendrogenesis; therefore, this upregulation of IGF-1 may contribute towards progesterone-induced promotion of OL proliferation and differentiation. Furthermore, DHT increases IGFBP-5 expression in human embryonic-derived NSCs [285], and both estradiol and DHT increase IGFBP-5 expression in the spinal cord of male mice [306]. Altogether, further work testing combinations of hormones is needed to better explore and define the complex relationships between hormonal systems and their effects on oligodendrogenesis.

Furthermore, in vivo work is limited, especially outside of disease contexts, and we have little understanding of whether and how hormones affect OPCs and OLs differently based on brain region, cellular age, or organismal age. In many cases, the in vivo experimental designs we describe utilized constitutive overexpression/deletion of hormones or receptors, which offer little temporal resolution and may be complicated by widespread alterations to the developmental trajectory of the organism. In addition, most of these manipulations were not restricted to OL lineage cells. Generating model organisms with genetic manipulations specifically within the various stages of the OL lineage would offer greater insight into direct hormonal modulation of oligodendrogenesis. Designing these manipulations to be temporally controlled would present the opportunity to test hormonal effects on oligodendrogenesis across the full extent of the lifespan, from early life to transitional periods such as puberty, and throughout adulthood and aging. Ultimately, understanding the direct vs. indirect effects of hormones on oligodendrogenesis in vivo will provide greater understanding not only of the mechanisms of hormonal action, but also of the suitability of hormonal interventions in providing direct, as opposed to off-target, effects in the CNS. 

Lastly, while we have detailed the effects of many different hormones on oligodendrogenesis, this is only a small fraction of the hormones that regulate development and adult plasticity. In the previous section, we discussed studies that focus on hormones that act in a paracrine fashion in the brain. However, many hormones remain to be explored. Our review has focused on the existing literature, but the absence of evidence does not imply that such hormones have no role in regulating oligodendrogenesis.

## 6. Conclusions

**Figure 2:**
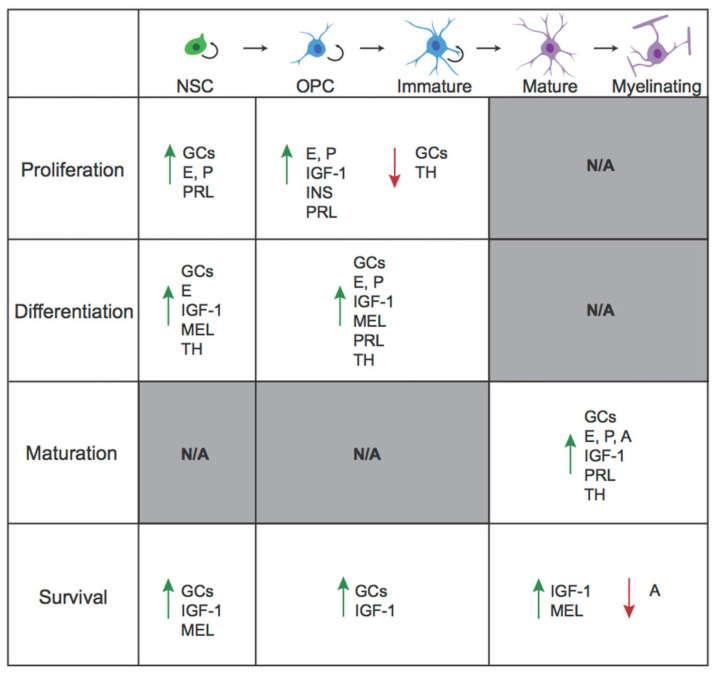
Oligodendrogenesis is differentially affected by various classes of hormones. Hormones can affect proliferation, differentiation, maturation, and survival across the OL lineage. Green arrow, promote; Red arrow, downregulate. Grey shaded sections indicate a process that is not applicable (N/A) at that cellular stage. Proliferation and differentiation only occur in the first three cellular stages, while maturation only occurs in the final stages. GC, glucocorticoids; E, estrogens; P, progestogens; A, androgens; IGF-1, insulin-like growth factor-1; INS, insulin; PRL, prolactin; MEL, melatonin; TH, thyroid hormones.

Hormones regulate nearly every stage of human development, and in adulthood, their levels can be modulated by a host of conditions, including stress, pregnancy, menopause, and aging. These hormonal fluctuations influence the brain and behavior, in part by altering the birth and development of new cells. As we have described in this review, hormonal modulation of plasticity extends beyond neurogenesis and into the realm of glia. Overall, it is clear that hormones across many classes exert robust effects on oligodendrogenesis, not only during development, but also in adulthood. Many of these hormones, including IGF-1, thyroid hormones, and the sex hormones, act to increase OPC differentiation and enhance the maturation of mature, myelinating OLs through both direct and indirect mechanisms (Figure 2). Clearer insight into the mechanisms governing hormonal regulation of oligodendrogenesis will enable better understanding of experience-dependent myelination in the human brain, and has important implications for myelin repair in a range of disorders, which we describe in our companion review in this issue [25]. 

## Abbreviations

A4	androstenedione
A5	androstenediol
AKT	protein kinase B
AMPA	α-amino-3-hydroxy-5-methyl-4-isoxazolepropionic acid
APC	adenomatous polyposis coli
AR	androgen receptor
BBB	blood–brain barrier
BrdU	bromodeoxyuridine
CNPase	2’,3’-cyclic-nucleotide 3’-phosphodiesterase
CNS	central nervous system
Cort	corticosterone
CreER	Cre recombinase/estrogen receptor fusion protein
CRH	corticotropin releasing hormone
Dex	dexamethasone
DHEA	dehydroepiandrosterone
DHT	dihydrotestosterone
Dio2	type 2 deiodinase
E2	17-β estradiol
ER	estrogen receptor
ERK	extracellular signal-regulated kinase
FGF2	fibroblast growth factor 2
GalC	galactosylceramidase
GC	glucocorticoid
GH	growth hormone
GPR	G-protein coupled receptor
GPDH	glycerol phosphate dehydrogenase
GST-pi	glutathione-S-transferase-pi
GR	glucocorticoid receptor
HSD	hydroxysteroid dehydrogenase
IGF-1	insulin-like growth factor-1
IGF1R	insulin-like growth factor-1 receptor
IGFBP	insulin-like growth factor binding protein
IR	insulin receptor
MAPK	mitogen-activated protein kinase
MBP	myelin basic protein
MEK	mitogen-activated protein kinase kinase
mPR	membrane-bound progesterone receptor
MT1	melatonin receptor 1
MT2	melatonin receptor 2
NDRG1	N-myc downstream-regulated gene 1
NE	norepinephrine
NG2	neural/glial antigen 2
NSC	neural stem cell
O-2A	bipotential oligodendrocyte-type 2 astrocyte progenitor cells
OL	oligodendrocyte
Olig1	oligodendrocyte transcription factor 1
Olig2	oligodendrocyte transcription factor 2
OPC	oligodendrocyte precursor cell
OPP	oligodendrocyte pre-progenitor
PACAP	pituitary adenylate cyclase activating peptide
PDGF	platelet-derived growth factor
PDGFRα	platelet-derived growth factor receptor alpha
PGRMC1	membrane-associated progesterone receptor membrane component 1
PI3K	phosphatidylinositol 3-kinase
PKA	protein kinase A
PLC	phospholipase C
PLP	proteolipid protein 1
PR	progesterone receptor
PRLR	prolactin receptor
PSA-NCAM	polysialylated-neural cell adhesion molecule
RXR	retinoid-X receptor
SGK1	serum glucocorticoid regulated kinase 1
T3	triiodothyronine
T4	thyroxine
TH	thyroid hormone
TR	thyroid hormone receptor
TRE	thyroid response element
VIP	vasoactive intestinal peptide
VPAC1	vasoactive intestinal peptide receptor 1
VPAC2	vasoactive intestinal peptide receptor 2
YFP	yellow fluorescent protein
